# Enhanced Neural Decoding With Optically Pumped Magnetometer MEG Using Multivariate Pattern Analysis

**DOI:** 10.1109/TNSRE.2026.3719472

**Published:** 2026-08-05

**Authors:** Jiawei Liang, Yulia Bezsudnova, Anna Kowalczyk, Yu Sun, Ole Jensen

**Affiliations:** Centre for Human Brain Health, School of PsychologyUniversity of Birmingham 1724 Birmingham B15 2TT U.K.; Southeast University–University of Birmingham Biomedical Engineering Joint Research Center Nanjing 210096 China; Department of Imaging NeuroscienceUniversity College London London WC1N 3AR U.K.; Laboratory for Imaging Science and Brain Health, School of Biological Science and Medical EngineeringSoutheast University Nanjing 210096 China; Department of Experimental PsychologyUniversity of Oxford 6396 Oxford OX1 3EL U.K.; Oxford Centre for Human Brain Activity, Oxford Centre for Integrative NeuroimagingDepartment of PsychiatryUniversity of Oxford Oxford OX3 9DU U.K.

**Keywords:** Brain–computer interfaces, magnetoencephalography (MEG), multivariate pattern analysis (MVPA), neural signal decoding, optically pumped magnetometer (OPM), spatial-frequency analysis

## Abstract

Multivariate pattern analysis (MVPA) provides a sensitive means to decode distributed neural activity from magnetoencephalography (MEG) by jointly exploiting temporal and spatial information. Optically pumped magnetometer (OPM)–based MEG enables close-to-scalp measurements and is therefore expected to capture neuromagnetic fields with higher spatial resolution compared to conventional systems based on superconducting quantum interference devices (SQUIDs). In this study, we conducted a within-subject comparison of OPM- and SQUID-MEG using time-resolved MVPA to decode neural responses to visual objects presented as images and corresponding words, recorded from the same participants under an identical experimental paradigm and matched preprocessing. To isolate the contribution of spatial sampling, decoding performance was evaluated under controlled sensor counts and spatial-frequency content, quantified using a spherical harmonic expansion of the sensor topographies. A complementary full-array SQUID analysis was also included as a reference. The results indicated that OPM-MEG achieved higher decoding accuracy than SQUID-MEG in the sensor-matched comparison, with the advantage being particularly evident for word decoding, where OPM also exceeded the full-array SQUID across a broad range of sensor counts. Further analysis of spatial-frequency content revealed that OPM-MEG benefited from the inclusion of higher-order spatial components. These findings demonstrate that OPM-MEG enhances the recoverability of fine-grained neural representations for multivariate decoding, with implications for cognitive neuroscience research and neural engineering applications such as brain–computer interfaces.

## I. Introduction

Multivariate pattern analysis (MVPA), which uses distributed patterns of neural activity to discriminate between experimental conditions or stimulus categories, has proven to be an invaluable tool across a wide range of cognitive neuroscience domains [1], [2], [3], [4], [5], [6], [7], [8] due to its ability to capitalize on both the spatial and temporal structure of brain activity [9], [10], [11], [12]. The multivariate approach is also applied in brain-computer interfaces (BCIs) [13], [14], [15], [16] including novel brain-to-text efforts [17], [18], [19]. Compared to a cryogenic magnetoencephalography (MEG) system based on superconducting quantum interference devices (SQUIDs), optically pumped magnetometers (OPMs) allow for a more flexible sensor arrangement and smaller distances from the sensors to the scalp, which result in higher recorded brain signals [20], theoretically improved spatial resolution [21], [22], and the potential for more anatomically tailored sensor coverage [23], particularly in regions like the frontal lobes that are traditionally challenging to access with SQUID systems [24]. These advantages over SQUID make OPM especially well-suited for studying spatially distributed brain processes via MVPA [25], [26]. The relevance of this question is not limited to technical benchmarking. Conventional ERP studies have shown that evoked visual components such as the N170 can index altered emotional, semantic, and social-cognitive processing in neuropsychiatric conditions, including bipolar disorder and ADHD [27], [28]. However, ERP analyses typically summarize activity at selected sensors and latencies, whereas MVPA exploits distributed spatiotemporal patterns and can reveal representational information that is not reducible to the amplitude or latency of a single component. Therefore, improved MEG sensitivity to stimulus-evoked multivariate structure may have broader implications for applied cognitive neuroscience and clinical research [4], [29]. The potentially finer spatial resolution of OPM system may also help improve the limited performance of MEG reported in a related study [30], [31]. Furthermore, since the OPM sensor array can be adapted to different head size across the life-span, it sets the stage for developmental studies using MVPA in children, infants and possibly even fetuses [32], [33], [34].

Although OPM sensors detect stronger neural signals, they exhibit higher levels of intrinsic noise compared to SQUID-based systems [35]. However, this limitation may be partly mitigated in the context of MVPA, because classifiers rely on distributed patterns across sensors rather than relying on the amplitude of individual channels [5]. Nevertheless, noise remains a limiting factor for decoding accuracy; our hypothesis is instead that sensors positioned closer to the scalp may allow OPM systems to capture larger task-related neural signals and thereby improve the effective signal-to-noise ratio. Moreover, this advantage should be more pronounced in signal components of higher spatial frequency, as the amplitudes decrease exponentially with the order of spherical expansion signals in the MEG sensor array [36, p. 41].

Furthermore, it remains to be examined how the number of MEG sensors affects the MVPA performance in our specific experiment. Spatial-frequency analyses of MEG/EEG indicate that sensor count directly governs the range of spatial frequencies that can be sampled [37].

To evaluate the performance of OPM-MEG in multivariate analysis, we here empirically test the efficacy of MVPA using SQUID and OPM data. Our paradigm includes two modalities: pictures and the corresponding written words (Fig. 1). The data were analyzed using pairwise decoding [4]. By directly assessing decoding performance across different modalities and conditions with controlled experimental paradigm and preprocessing pipeline, we aimed to determine whether and how the robustness and spatial advantages of OPMs translate into measurable improvements in neural decoding.

**Fig. 1.**
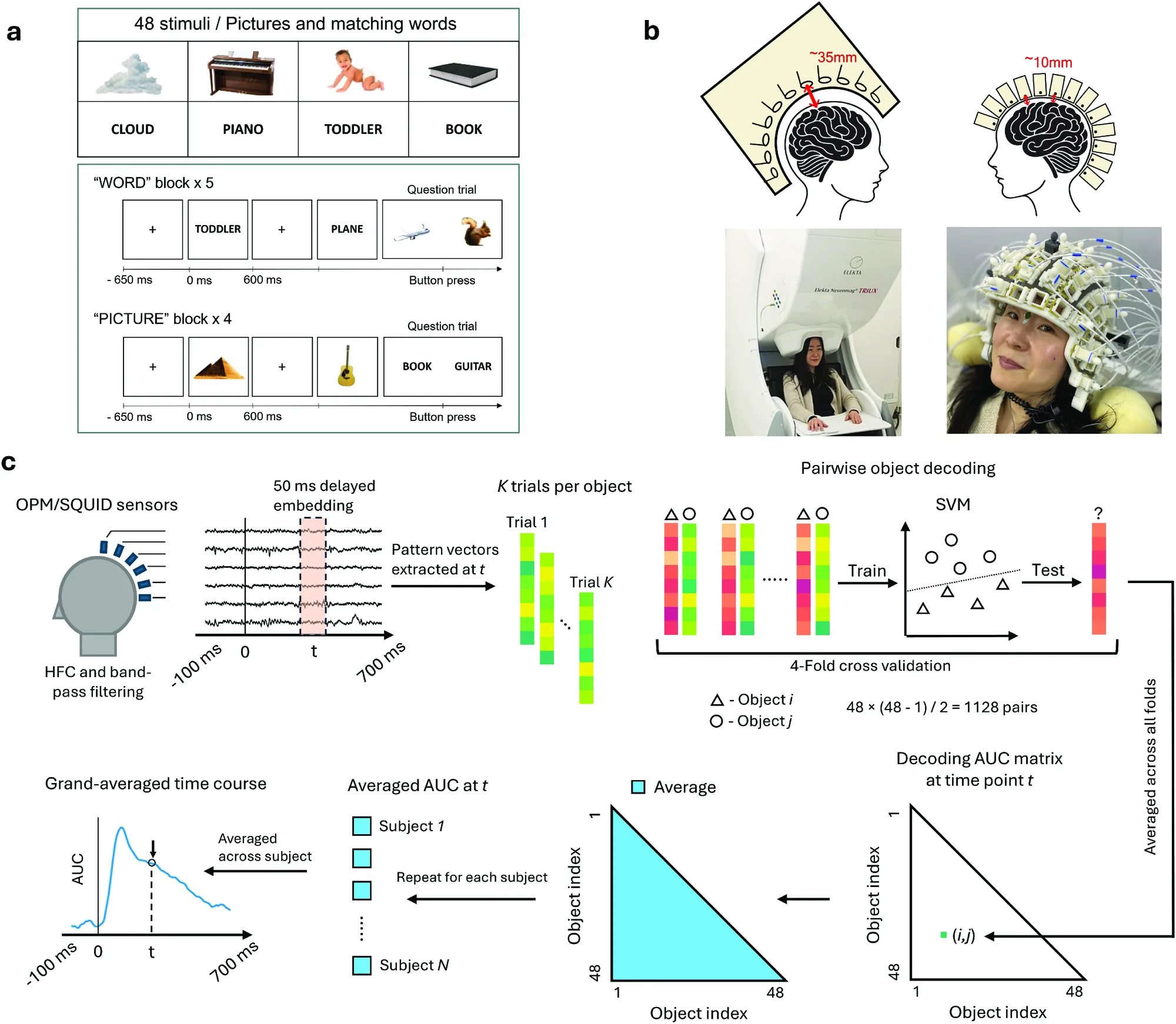
**Experimental design and multivariate analysis.** (a) Experiment paradigm. The stimulus set consists of 48 pictures and corresponding words in text. Five blocks for word stimulus and four for picture were arranged alternately. Within each block, each stimulus was presented in random order and repeated for 4 times, with one time followed by a question trial in the opposite modality. The stimulus duration was 600 ms and the fixation duration was randomly *550 ∼  750* ms. (b) Demonstration of the MEGIN Elekta SQUID system (left) and FieldLine HEDscan OPM system (right). The indicated values denote approximate sensor-to-scalp separations and should not be interpreted as distances to cortical sources, which span both gyral and sulcal surfaces. Images are reproduced with participant’s written consent. (c) MVPA pipeline of pairwise object decoding. Epochs were delimited 100 ms before and 700 ms after the stimulus onset from pre-processed OPM/SQUID recordings, yielding 16 trials for each picture and 20 for each word. For each timepoint, sensor space pattern vectors were extracted with 50 ms time-delayed embedding. For each of the total 1128 object pairs, a binary SVM classifier was trained and evaluated via 4-fold cross validation. The decoding accuracy was averaged across all conditions and participants at each time point, which ultimately produced the AUC time course for picture and word modalities.

## II. Materials and Methods

### A. Experimental Paradigm

The experiment paradigm was adapted from a previous study [38], with some stimuli replaced and the task slightly modified (Fig. 1a). Visual stimuli of 48 objects were presented in the form of pictures and the corresponding written words (font: Arial, bold, 75). The stimulus measured *400× 400* pixels and subtended a visual angle of 6°, which was projected on a screen with grey background. Projectors of identical model (VPixx PROPixx) were used in OPM and SQUID sessions both operating at 120 Hz refresh rate.

There were nine blocks in the experiment (five blocks for words and four blocks for pictures). Within each block, the participant viewed a series of stimuli for 600 ms each, which were separated by fixation crosses for a random duration of 550 to 750 ms. There were randomly inserted questions on average every fifth trial that asked the participant to choose the last seen stimulus from two options presented in the opposite modality of that block. These questions were included to keep participants attentive, and responses were made via button press within a timeout of 3.5 s. Participants were allowed to take self-paced breaks between blocks, ranging from at least 5 s to a maximum of 300 s.

### B. Experiment Participants

Twelve healthy adults participated in the study. Three participants were excluded due to data quality issues: one associated with OPM trigger failure, one with an interrupted OPM recording, and one showing abnormal noise patterns in the SQUID PSD. Finally, nine participants (mean age = 25.1 +/- 3.1 years old; 6 female) were included in consequent analysis. The order in which participants underwent OPM and SQUID sessions was counterbalanced. All participants were native English speakers with normal or corrected-to-normal vision.

The study was conducted at the Centre for Human Brain Health at the University of Birmingham. The study was approved by University of Birmingham Ethics Committee (ERN_18-0226AP34). The participants provided written informed consent and received compensation of £15 per hour for their participation.

### C. MEG Acquisition

SQUID data were acquired using a 306-channel TRIUX MEGIN system (Fig. 1b, left) consisting of 102 sensor locations. Each location contains one single-axis radial magnetometer and a pair of orthogonal planar gradiometers, yielding 102 magnetometer channels and 204 gradiometer channels in total. An online band-pass filter from 0.1 to 330 Hz was applied before the data were sampled at 1000 Hz.

**Fig. 2.**
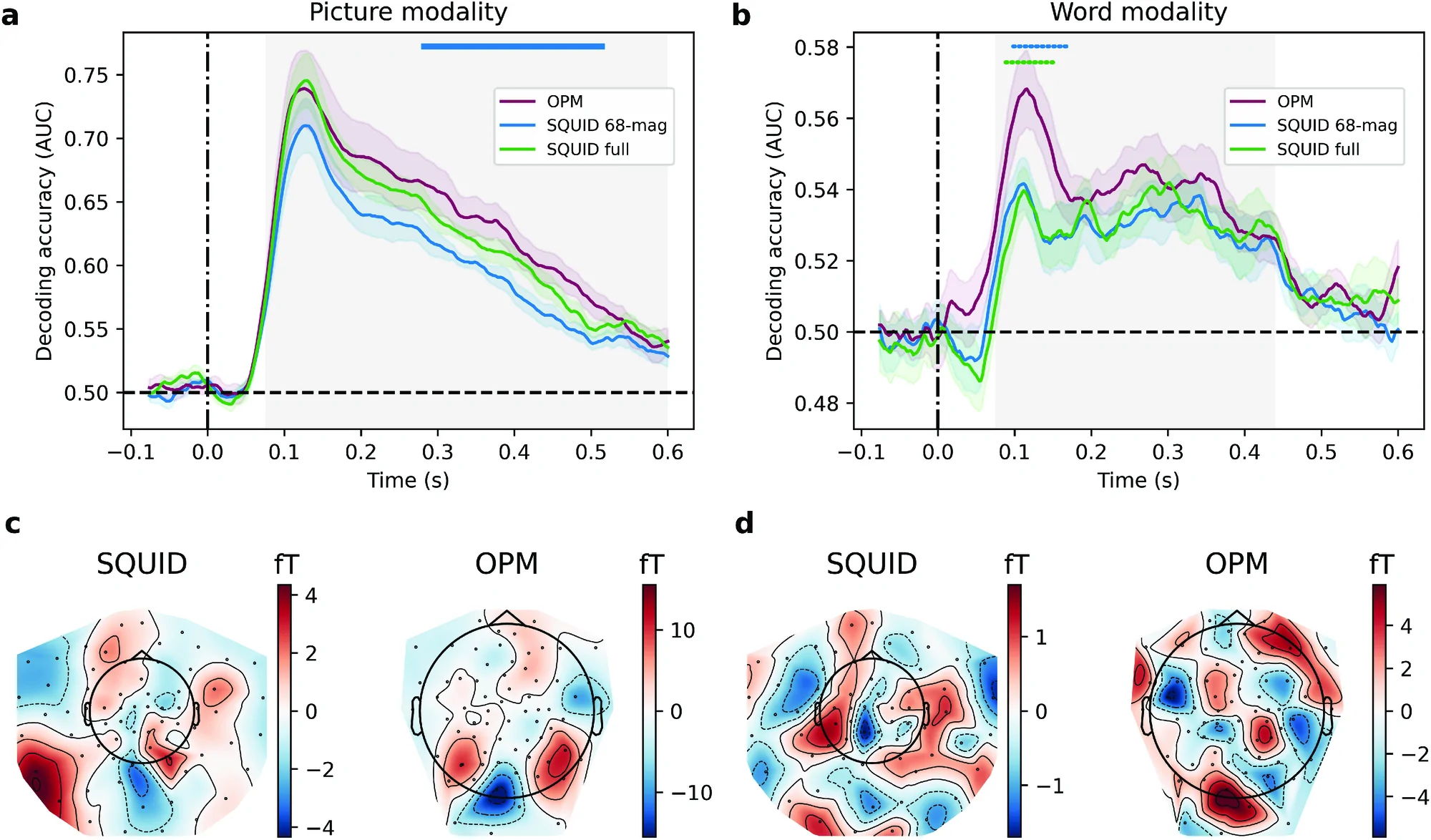
**Time-resolved multivariate pairwise object decoding.** (a)(b) Time-resolved decoding AUC for picture and word modalities. Purple line, OPM; blue line, SQUID 68-magnetometer subset; green line, SQUID full array. Shaded error bands indicate the standard error of the mean across participants. The horizontal dashed line marks chance-level performance (AUC = 0.5), and the vertical dash-dotted line marks stimulus onset. Horizontal bars indicate clusters from paired, one-tailed cluster-based permutation tests comparing OPM with the SQUID 68-magnetometer subset (blue) and SQUID full array (green), corrected for multiple comparisons over time. Solid bars denote significant clusters ( ${p} < $.05), and dashed bars denote trend-level clusters (. $05< {p} < $. 10). The light grey shaded regions indicate predefined effective decoding intervals: 75–600 ms for pictures and 75–440 ms for words used to compute averaged decoding accuracy in the subsequent analysis. (c)(d) Topographical activation patterns at peak decoding times (127 ms for pictures; 114 ms for words) derived from classifier weights, averaged across participants, stimulus objects and time points of delayed embedding.

OPM data were recorded with a FieldLine HEDscan system (Fig. 1b, right) with 73 single-axis SERF-OPM sensors operating at 5000 Hz sampling rate with an online low-pass filter at 500 Hz. Seventy-two OPM sensors were uniformly distributed across the helmet with 144 mounting slots, covering the whole head. One sensor was used as eyeblink detector. A few sensors randomly failed or suffered from high noise level, which were disabled before recording.

In both systems, the sensor geometry used for preprocessing was derived from the corresponding manufacturer-defined helmet setup. For the SQUID system, sensors are fixed by the rigid TRIUX helmet geometry. For the OPM system, sensor positions and orientations were obtained from the HEDscan Smart Helmet, which provides automatic sensor localization, and were recorded in the acquisition files.

Although residual uncertainty in the sensor geometry may affect the accuracy of environmental-field modelling, this uncertainty is expected to have a stronger impact on source-localization analyses than on the present sensor-space MVPA comparisons.

### D. Data Pre-Preprocessing

The data were analyzed using the open-source toolbox MNE Python v1.7.1 [39] following the standards defined in the FLUX Pipeline [40]. A two-step sensor dropping method was applied to OPM data at start. First, the sensors with excessive broad-band noise in the raw power spectral density (PSD) were visually identified and rejected. Second, a homogeneous field correction (HFC; order = 2) filter [41], [42] was applied to help identify additional sensors with persistent abnormal PSD profiles. This preliminary HFC was applied only to a temporary copy of the data for inspection, and sensors that still showed abnormal PSD characteristics were further excluded. This resulted in an average of 69.8 sensors (a minimum of 68 sensors). Finally, unprocessed raw data were retrieved from the remaining sensors for the following steps.

The primary comparison between OPM and SQUID MEG was based on matched sensor counts. To normalize the number of sensors across systems and participants, SQUID magnetometers and OPMs were subsampled to a maximum of 68 (and fewer, as described in the spatial property analysis section). In addition to this matched-sensor analysis, we also retained the full SQUID array as a reference condition. Subsampling was performed using random k-means clustering in the sensor-layout space to maintain approximately even coverage of the helmet. For each target sensor number, the projected sensor-layout coordinates obtained from MNE were clustered into k groups using the standard k-means objective, which minimizes the within-cluster sum of squared Euclidean distances between sensor positions and their assigned centroids. The sensor closest to each centroid was then selected for the subsampled array. This procedure was intended to avoid spatial bias and to assess the dependence of decoding performance on sensor count, rather than to optimize decoding performance or target any specific cortical region.

For each subsampled sensor set, the corresponding raw data were extracted independently, and the full preprocessing pipeline was then applied separately. Specifically, an HFC filter (order = 2) was applied to the subset of MEG sensors to model and remove low-order environmental field components. The data was further low pass filtered at 200 Hz and then down sampled to a sampling rate of 500 Hz. Notch filters at 50 Hz and 100 Hz were used to remove line noise. A band-pass filter between 1 to 100 Hz was applied to reduce low frequency fluctuations introduced by lift operation nearby and other high frequency noise. The paradigm events were annotated based on the trigger channel information.

### E. Pairwise Object Decoding

As shown in Fig. 1c, the MEG data acquired with SQUID and OPM systems were first segmented into trials (−100 to 700 ms) that were time-locked to the stimulus onset. All the N sensor values were further concatenated along a sliding time window of ±25 ms (25 time points in total), e.g. resulting in a N *× 25*-dimensional feature vector for each time point reflecting the N sensors.

MVPA was applied to the processed MEG data to decode the object identity pairwise, separately for each participant’s OPM and SQUID data. First, the feature vectors associated with all trials of each object in the paradigm were extracted, yielding 16 trials per picture and 20 trials per word. For each stimulus modality, binary classification of object pairs was performed at each time point using a support vector machine (SVM) with four-fold cross-validation, chosen so that the number of trials was evenly divisible by the folds. Within each fold, features were z-scored to zero mean and unit variance prior to classification. Decoding performance was quantified using the area under the curve (AUC) averaged across all folds.

For each participant and MEG system, the aforementioned steps resulted in the strictly lower triangular part of a 48 by 48 representational dissimilarity matrix of the pairwise object decoding at each time point. The decoding AUC was further averaged across all object pairs (n = 1128) and participants (n = 9) at each time point, ultimately resulting in AUC time courses for picture and word modalities.

To investigate the spatial distribution of brain activity underlying the decoding, the topographical activation patterns on the OPM and number-matched SQUID sensor arrays were extracted using back-projection of classifier weights via the method described in [43]. Only radial magnetometers of both systems were included in this analysis.

### F. Spatial Sampling and Spatial Frequency Analysis

To evaluate the SQUID and OPM systems, we first subsampled the sensors from 10 to 68 and repeated the preprocessing and MVPA decoding pipeline for each sensor count. The averaged decoding AUC over time was then calculated as a function of the number of sensors.

Complementary to this sensor-count analysis, we also examined the contribution of signal components at different spatial frequencies. For this analysis, we used a Signal Space Separation (SSS)-derived [36], Adaptive Multipole Modeling (AMM)-style [44] projection based on the spherical-harmonic field expansion implemented in MNE-Python. Since higher harmonic orders represent higher spatial frequency components of the modelled brain signal [45], [46], we progressively increased the internal expansion order from 1 to 7. The maximum order was constrained by the 68 available sensors [46], and the external harmonic order was fixed at 2 to match the order used for HFC in the previous analysis.

To quantify how rapidly decoding performance saturated as higher-order spatial components were included, we fitted the AUC-versus-order curve for each participant, modality, and MEG system with a three-parameter sigmoid function. The fitted curves are shown in Fig. 4 to visualize the relationship between harmonic order and decoding performance:

\begin{equation*} F\left ({ L }\right) =\frac {a}{1+e^{-k\left ({ L-L_{0} }\right)}} \tag {1}\end{equation*}

where *L* denotes the maximum order of harmonics, *k* the steepness of the function, *a* the upper limit and $L_{0}$ the middle point. The fitted steepness parameter *k* was then used as a summary measure of how strongly decoding performance was driven by lower-order versus higher-order spatial components. A larger *k* indicates that decoding performance increases rapidly with lower spatial frequency components and saturates with higher frequencies, whereas a smaller *k* indicates a more gradual increase across orders and greater reliance on higher-frequency components.

**Fig. 3.**
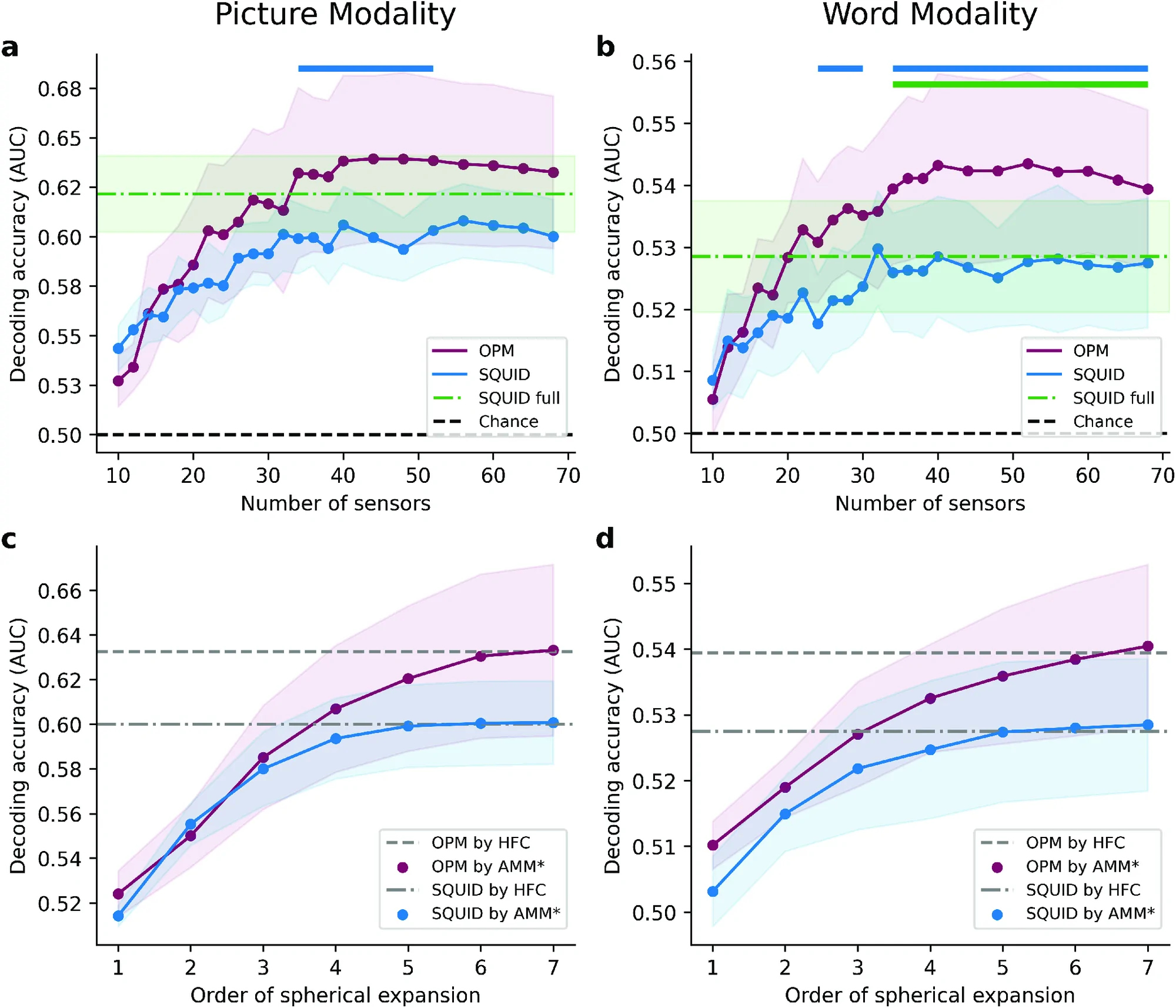
**MVPA decoding performance as a function of the number of sensors and the order of spherical expansion.** (a)(b) Mean decoding AUC (averaged across 75–600 ms for pictures; 75–440 ms for words) with different numbers of sensors. Purple line, OPM; blue line, SQUID; green dash-dotted line, SQUID full array; black dashed line, chance level (AUC = 0.50). Shaded areas represent the 95% confidence interval (CI) across participants. Horizontal bars above the curves indicate significant clusters from paired, one-tailed cluster-based permutation tests over sensor count, corrected for multiple comparisons. Blue bars denote sensor-count ranges in which OPM outperformed the matched SQUID subset, and green bars denote ranges in which OPM outperformed the SQUID full array ( ${p} < $. 05). (c)(d) Mean decoding AUC with the maximum order of spherical harmonics included. Colored lines represent results obtained using the SSS-derived, AMM-style filtering (shaded areas for 95% CI). Dashed and dash-dotted lines indicate decoding AUC obtained after applying HFC filtering to the MEG data, serving as reference levels for OPM and SQUID respectively.

### G. Statistical Analysis

To control for multiple comparisons over time-points when comparing the classification results, a non-parametric cluster-based permutation test [47], [48], [49] was applied. A cluster-forming threshold corresponding to *p < *.05 (one-tailed) was applied at the sample level. Cluster-level significance was assessed against a one-tailed null distribution of the maximum cluster statistics obtained from 10000 random sign-flip permutations, and clusters with *p < *.05 were considered significant. To evaluate the differences in the growth trends of decoding AUC with harmonic order between OPM and SQUID data, a Wilcoxon signed-rank test was conducted to compare the fitted steepness k between the two systems. Fits with an adjusted $R^{2}$ below 0.8 were excluded from statistical testing as outliers.

## III. Results

We conducted a within-participant comparison of MEG signals acquired with a conventional SQUID system and a single-axis OPM system, with MVPA applied to a visual object and word paradigm (Fig. 1a). Participants responded accurately to the attention-check questions (OPM: mean (M) = 94.5%, standard deviation (SD) = 5.40%; SQUID: M = 96.5%, SD = 2.35%). A Wilcoxon signed-rank test revealed no significant difference in accuracy across MEG systems ( *V =15*, *p =*. 43), indicating that participants maintained comparable levels of attention throughout the task.

The MEG data were preprocessed and time-resolved object decoding accuracy (AUC) for each modality was quantified. To further assess spatial properties, we systematically varied the number of sensors included in the analysis and examined the contribution of different spatial frequency components of the measured signals using a spherical expansion approach. The following sections present the results of these analyses.

### A. Time-Resolved AUC of Pairwise Object Decoding

The MVPA approach yielded a decoding AUC time course of each pair of visual stimuli for each participant. A grand average curve was computed across all object pairs. Then these grand averages were aggregated across participants to assess the comparative performance of the OPM and SQUID based MEG systems for the picture and word modalities.

We observed robust decoding for both pictures and words across OPM, the SQUID 68-magnetometer subset, and the SQUID full array. The overall profiles and value ranges of the AUC time course are in agreement with the findings of previous MVPA studies involving pictures and written or spoken words as stimuli [10], [38], [50]. As shown in Fig. 2a and b, the temporal patterns appeared similar between OPM and SQUID, but the relative advantage of OPM differed between modalities. For pictures, OPM showed higher decoding accuracy than the SQUID 68-magnetometer subset during 278-518 ms window ( *p =*.020, cluster permutation), whereas no cluster reached the p <.10 threshold when OPM was compared with the SQUID full array. For words, OPM showed a trend-level advantage over the SQUID 68-magnetometer subset during 98-168 ms interval ( *p =*.057, cluster permutation), and a similar trend-level cluster was also observed relative to the SQUID full array during 88–154 ms ( *p =*.072). These results suggest that the OPM advantage was expressed differently across modalities: in the picture modality it was primarily evident under the sensor-matched comparison, whereas in the word modality a comparable early trend was present relative to both the SQUID 68-magnetometer subset and the SQUID full array.

The activation topographic maps were computed at the midpoint between the peak AUC latencies of SQUID and OPM for each modality to visualize the spatial patterns underlying decoding. The sensors contributing most strongly to picture-object decoding were predominantly located over occipital areas for both OPM and SQUID (Fig. 2c), whereas for word stimuli the contributing sensors showed a more dispersed distribution across parietal, temporal, and frontal areas (Fig. 2d). To account for participant-specific adjustment of OPM sensor positions, the average topographic maps were derived through clustering and interpolation across all participants’ sensor layouts.

Together, these results show that both systems produced robust decoding consistent with prior MVPA studies, with OPM showing systematically higher accuracy. We next examined how this advantage depends on sensor number and spatial frequency content.

### B. Number of Sensors

We then repeated the MVPA considering a subsampling of the number of sensors from 10 to 68. Averaged decoding performance was computed within predefined time windows, 75–600 ms for pictures and 75–440 ms for words relative to stimulus onset as indicated by the shaded intervals in Fig. 2a and b and based on previous reports [4], [10], [50]. Fig. 3a and b illustrate the averaged AUC as a function of the number of subsampled sensors for the picture and word modalities, respectively. The confidence intervals reflect inter-participant variability. To provide an additional reference, the averaged decoding performance of the SQUID full array is also shown as a horizontal dash-dotted line with its confidence interval.

**Fig. 4.**
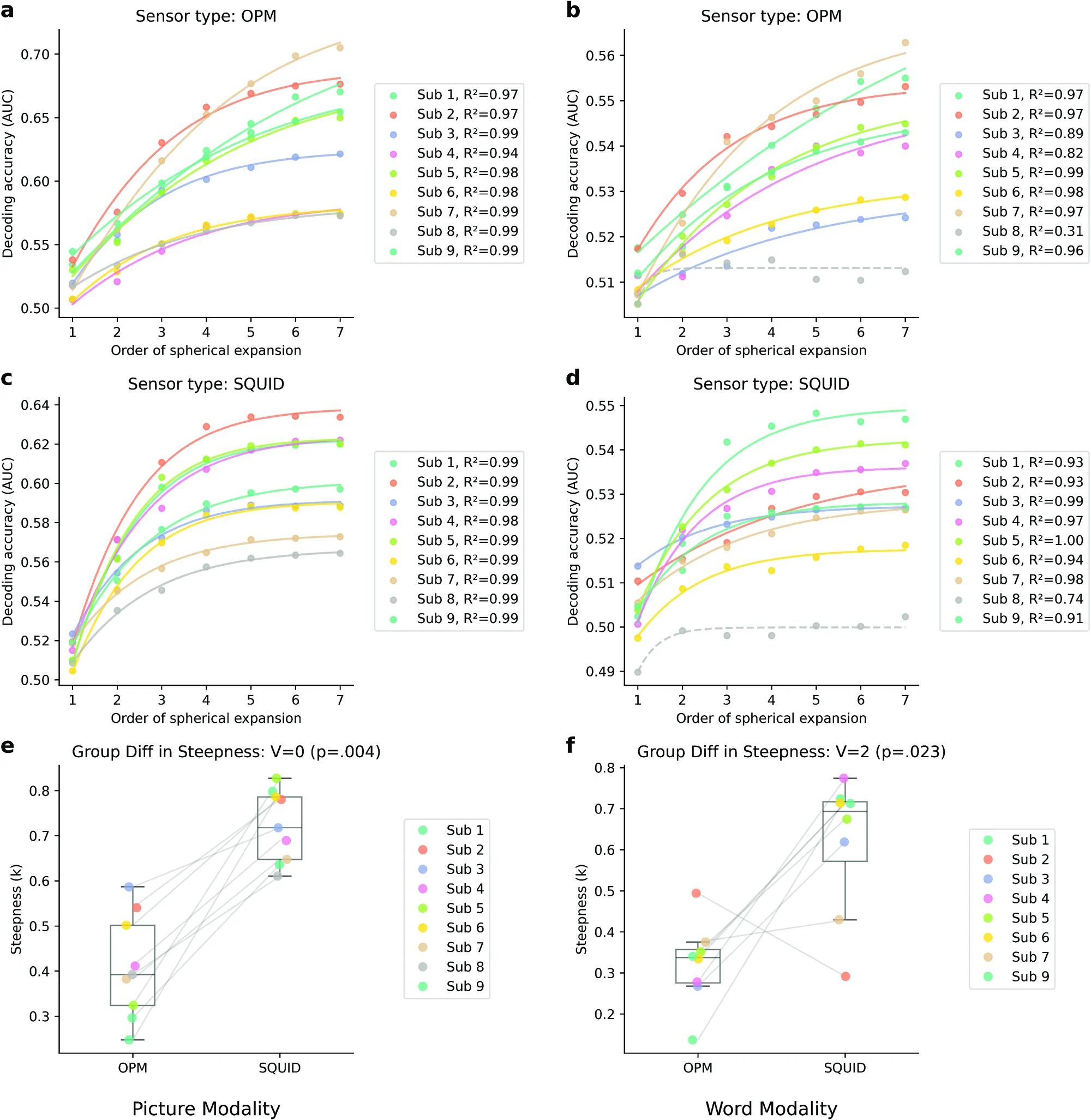
**Individual decoding performance and sigmoid fits across orders of spherical expansion.** Decoding accuracy (AUC) as a function of spherical harmonic expansion order of OPM (a)(b) and SQUID (c)(d) signals for the picture and word modalities, respectively. Data from individual participants (coloured dots) were fitted with a three-parameter sigmoid function (solid lines). Adjusted ${R}^{{2}}$ values indicated good model fits across participants, except for one outlier in the word modality (OPM: ${R}^{{2}} =0.31$; SQUID: ${R}^{{2}} =0.74$), which was excluded from group-level analysis for that modality. (e)(f) Boxplots show the distribution of fitted steepness parameter $\boldsymbol {k}$ across participants, with paired individual data points overlaid. Steepness was significantly lower for OPM compared to SQUID in both picture (Wilcoxon signed-rank test, ${V} =0$, ${p} =$.004) and word modalities ( ${V} =2$, ${p} =$.023).

For both modalities, MVPA performance increased from 10 to ~30 sensors and plateaued after ~40 sensors. After reaching this plateau, decoding performance showed only modest fluctuations at higher sensor counts. Compared with the matched SQUID subset, OPM yielded higher decoding accuracy, with significant clusters at 34–52 sensors for pictures ( *p =*.033) and at 24–30 and 34–68 sensors for words ( *p =*.035 and *p =*. 008, respectively). When compared with the SQUID full array, OPM did not show a significant cluster for pictures, whereas for words OPM outperformed the full array across 34–68 sensors ( *p =*.012). In summary, decoding performance saturated with approximately 40 sensors, and OPM achieved higher accuracy than the matched SQUID subset, reaching comparable performance with fewer sensors. This advantage was particularly evident for the word modality, where OPM also exceeded the SQUID full-array reference across a broad range of sensor counts.

### C. Spatial Frequency Components

Next, we examined the accumulative contribution to MVPA performance considering the spatial frequency components in the MEG signals. This analysis used the same predefined time windows as the sensor-number analysis, namely 75–600 ms for pictures and 75–440 ms for words relative to stimulus onset. Fig. 3c and d show the decoding AUC as a function of the order of spherical expansion using the SSS-derived, AMM-style filtering. The AUC from data pre-processed using HFC is presented as a reference where the inner spatial components were not limited. For the picture modality, OPM and SQUID MEG data exhibited comparable increase in decoding performance using the spherical harmonics from 1 to 3. While the AUC of SQUID plateaued at order 5, the OPM performance improved until order 6. For the word modality, the performance was better for OPMs compared to SQUID and this advantage tended to increase as higher-order harmonics where included.

We next examined the spherical expansions for each participant. As shown in Fig. 4a-d, the data were generally well fitted by the three-parameter sigmoid function (adjusted $R^{2} > 0.9$ for most participants), except for one participant whose parameter estimate was constrained at the bound for word modality, yielding low goodness-of-fit ( $R^{2} =0.31$ for OPM and 0.74 for SQUID). This participant was therefore excluded from statistical testing for words. Fig. 4e and f show the group difference in steepness *k* between OPM and SQUID. OPM exhibited significantly lower steepness than SQUID for both pictures ( *V =0*, *p =*.004) and words ( *V =2*, *p =*.023), reflecting a more gradual increase that extended into higher-frequency components.

Taken together, the findings indicate that OPM decoding continued to benefit from higher-order spatial components, whereas SQUID performance saturated earlier, suggesting that OPM captures finer spatial details of the neural signal.

## IV. Discussion

In this study, we compared the performance of multivariate-pattern analyses from an OPM and a SQUID MEG system in terms of discriminating different visual stimuli of pictures and different words. We found that our OPM outperformed the SQUID MEG system in general. The advantage of OPM remained evident when we subsampled the sensors and it attained the plateau performance of SQUID with much fewer sensors (Fig. 3a and b). Furthermore, we studied how the decoding accuracy scaled with the spatial frequency content of the signal being decoded. The decoding accuracy plateaued for SQUID at lower spatial frequencies while OPM achieved a performance gain from higher-order spatial components (Fig. 4).

The AUC time course revealed a sustained advantage of OPM over SQUID throughout the decoding interval (Fig. 2a and b). The decoding peak occurred at similar latencies for both systems; however, OPM maintained a clear advantage even at the peak time for the word modality, where the difference in AUC between OPM and SQUID was greatest at the peak. These findings highlight two aspects of OPM’s superiority: a sustained decoding advantage across the entire decodable interval, which enhances sensitivity to the temporal dynamics of sensory processing; and an increased peak decoding capacity, which is particularly relevant for research- and clinically oriented BCI studies that exploit the strongest representational windows.

Whereas previous simulation studies based on spatial sampling and source localization analyses [37], [51] suggested that substantially larger numbers of sensors may be beneficial for adequately sampling neuromagnetic fields under typical MEG paradigms, our subsampling analysis indicated that the performance of object decoding plateaued around 40 sensors for both OPM and SQUID magnetometers (Fig. 3a and b). This discrepancy likely reflects the different goals of the analyses. The observed plateau therefore suggests that, for the present object-decoding task, most relevant information was captured by a moderate number of appropriately distributed sensors, rather than implying sufficiency for general-purpose OPM-MEG or source localization. Moreover, when the number of sensors must be reduced, the decline in MVPA performance will not appear as fast. Remarkably, even with fewer than 30 sensors, OPM still achieved decoding performance comparable to the full-array SQUID system, demonstrating its potential to facilitate wearable and scalable BCI-oriented research settings where the number of sensors is constrained [52], [53]. Further work is needed to determine whether this advantage of OPM generalizes to source localization or other MEG analysis approaches.

Our analysis of the relationship between order of spherical expansion and decoding performance provided converging evidence for a spatial-frequency-related advantage of OPM (Fig. 3cd, Fig. 4). At equivalent sensor densities, OPM benefited more from higher-order spatial components than SQUID did. This is consistent with the theoretical prediction that the amplitude coefficients of spherical harmonics decay with distance r according to $r^{-\left ({ l+2 }\right)}$ [36, p. 41], where *l* is the harmonic order. Thus, sensors placed closer to the scalp capture stronger signals, and the relative gain becomes even larger at higher orders. Importantly, this interpretation is not inconsistent with the plateau observed in the sensor-number analysis, because sensor count and spherical-harmonic order are not equivalent measures of spatial dimensionality. A spatially subsampled array may still retain higher-order field structure within its multivariate sensor pattern, even if adding more physical sensors provides limited additional benefit for cross-validated decoding. In summary, the closer sensor proximity in OPM yields an amplitude boost that is particularly pronounced for high spatial frequency components, enabling OPM to leverage them effectively despite its higher noise level.

When comparing the picture and word modalities, we first observed that decoding AUC was substantially lower for the word modality. This difference was also reflected in the topographic maps of decoding activation averaged over all participants, where word-related responses showed lower amplitudes (Fig. 2c and d). By contrast, the relative advantage of OPM over SQUID was more pronounced in the word modality. This was manifested as a sharper decoding peak (Fig. 2b), as well as clearer AUC differences emerging even with fewer sensors (Fig. 3b) or at lower orders of spherical expansion (Fig. 3d). These results suggest that OPM retains sufficient sensitivity to capture meaningful representations even when the overall neural signal is weaker. Consequently, OPM may offer particular benefits in research topics where signal strength is expected to be low, such as semantic processing, memory reactivation, or other higher-order cognitive functions.

Although the absolute improvement in decoding performance was modest, ranging from approximately 0.01 to 0.03 in AUC, it nevertheless suggests a consistent increase in the separability of condition-specific neural patterns under single-trial decoding. In addition, its practical relevance lies in combination with the sensor-subsampling results: OPM-MEG achieved comparable or superior decoding performance with fewer sensors, which may be advantageous for applied studies requiring wearable, flexible, or patient-tolerant recordings. Such features are particularly relevant for clinical and developmental populations, where comfort, head movement, and individualized sensor placement can strongly affect data quality.

Several limitations of the present study should be noted. First, our OPM system employed single-axis sensors rather than dual-axial or tri-axial ones. In the meanwhile, multi-axial OPM sensors are becoming increasingly available [54], [55], and recent developments have demonstrated OPM-MEG systems with up to 128 tri-axial sensors (384 channels) [56]. Such configurations may further facilitate noise reduction for OPM [46], [57] and possibly also contribute to better multivariate decoding. Therefore, the current single-axis setup should be regarded as an early-stage implementation rather than an upper bound on OPM-MEG performance. Future work should directly evaluate whether the advantage of OPM over SQUID generalizes or even amplifies with multi-axial and higher-density configurations. Second, our analysis focused exclusively on object-level decoding and did not examine higher-order semantic representations due to the small number of participants. While OPM demonstrated a clear advantage in distinguishing visual objects, it remains to be determined whether this advantage extends to more abstract decoding tasks, such as semantic category or conceptual processing. Third, the current paradigm was restricted to visual modality. Extending OPM-based MVPA to other domains, such as auditory processing [58], language, or motor actions, would provide a more comprehensive assessment of its advantages over SQUID.

The study included nine participants. While this provides adequate power to observe large effects in the MVPA comparison between OPM- and SQUID-MEG, increasing the sample size would improve sensitivity to nuanced patterns, refine effect-size estimates, and enhance generalizability. The current sample size and sex distribution also precluded a reliable assessment of whether the relative OPM–SQUID decoding difference was influenced by participant anatomy, such as head size or helmet fit. Future studies with larger and more balanced samples, together with explicit measurements of sensor-to-scalp distance and helmet fit, will be needed to examine this issue directly.

## V. Conclusion

In conclusion, our findings demonstrate that OPM–based MEG provides enhanced access to fine-grained neural information that can be effectively exploited by multivariate pattern analysis of visual processing compared to our SQUID system. This advantage was reflected not only in higher decoding performance, but also in the spatial-frequency analysis, which showed that higher-order spatial components contributed more strongly to OPM-based decoding. These results suggest that the closer proximity and flexible sensor placement of OPMs may improve access to spatially detailed, task-relevant field patterns, thereby enhancing the discrimination of visual stimuli with MVPA.

While much of the recent discussion on OPMs has emphasized their potential for improved source modelling [22], [23], [51], [59], our results highlight that enhanced multivariate decoding may be an equally important advantage. This suggests that OPM is not only valuable for precise localization but also for extracting distributed representational patterns that underpin modern cognitive neuroscience. Furthermore, the demonstrated robustness of OPM with reduced sensor density sets the stage for applying MVPA in populations such as infants and children, where small helmet sizes limit the number of sensors that can be accommodated. More broadly, the portability and flexibility of OPM systems open the door to studying cognition in more naturalistic and ecologically valid environments, and to extending brain–computer interface research to scenarios where lightweight and adaptable MEG solutions are needed. Taken together, these advances point toward a future where OPM-MEG expands both the methodological and conceptual horizons of neuroscience research.
